# Enteral Nutrition in Pancreaticoduodenectomy: A Literature Review

**DOI:** 10.3390/nu7053154

**Published:** 2015-04-30

**Authors:** Salvatore Buscemi, Giuseppe Damiano, Vincenzo D. Palumbo, Gabriele Spinelli, Silvia Ficarella, Giulia Lo Monte, Antonio Marrazzo, Attilio I. Lo Monte

**Affiliations:** 1 School of Oncology and Experimental Surgery, University of Palermo, via Del Vespro 129, 90127 Palermo, Italy; 2 Department of Surgical, Oncological and Dentistry Studies, University of Palermo, via Del Vespro 129, 90127 Palermo, Italy; E-Mails: vincenzopalumbo84@libero.it (V.D.P.); gabriele.spinelli@unipa.it (G.S.); silvia.ficarella@gmail.com (S.F.); antonio.marrazzo@unipa.it (A.M.); attilioignazio.lomonte@unipa.it (A.I.L.M.); 3 “P.Giaccone”, University Hospital, School of Medicine, School of Biotechnology, University of Palermo, via Del Vespro 129, 90127 Palermo, Italy; E-Mail: giuse.damiano@gmail.com; 4 School of Surgical Biotechnology and Regenerative Medicine in Organ Failure, University of Palermo, via Del Vespro 129, 90127 Palermo, Italy; 5 School of Biotechnology, University of Palermo, via Del Vespro 129, 90127 Palermo, Italy; E-Mail: giulialom@virgilio.it

**Keywords:** Enteral nutrition, pancreaticoduodenectomy, delayed gastric emptying, postoperative pancreatic fistula, postpancreatectomy hemorrhage

## Abstract

Pancreaticoduodenectomy (PD) is considered the gold standard treatment for periampullory carcinomas. This procedure presents 30%–40% of morbidity. Patients who have undergone pancreaticoduodenectomy often present perioperative malnutrition that is worse in the early postoperative days, affects the process of healing, the intestinal barrier function and the number of postoperative complications. Few studies focus on the relation between enteral nutrition (EN) and postoperative complications. Our aim was to perform a review, including only randomized controlled trial meta-analyses or well-designed studies, of evidence regarding the correlation between EN and main complications and outcomes after pancreaticoduodenectomy, as delayed gastric emptying (DGE), postoperative pancreatic fistula (POPF), postpancreatectomy hemorrhage (PPH), length of stay and infectious complications. Several studies, especially randomized controlled trial have shown that EN does not increase the rate of DGE. EN appeared safe and tolerated for patients after PD, even if it did not reveal any advantages in terms of POPF, PPH, length of stay and infectious complications.

## 1. Introduction

Gastrointestinal surgery involving intestinal resection and anastomosis often requires a period of starvation or “nil by mouth”, while new anastomosis heal. The aim of this strategy is to allow time for intestinal motility to return to normal, and to protect anastomosis from the stress of introducing oral fluids and diet [1]. Hepatobiliary surgery is usually performed in malnourished patients, and this condition if severe can be associated with a higher incidence of complications [2,3]. Pancreaticoduodenectomy (PD) is considered the gold standard treatment for periampullary carcinomas. This procedure is one of the most invasive operations in abdominal surgery and postoperative morbidity ranges between 30% and 40% [4]. PD results in a loss of gastric pacemaker and a partial pancreatic resection, and leads to a high incidence of postoperative malnutrition. The surgical trauma increases immune system suppression. The surgical injury as well as the malnutrition can provide bad postoperative outcomes. The malnutrition is worse in the early postoperative days, affects the process of healing, the intestinal barrier function and the incidence of postoperative complications [5,6]. Perioperative nutrition supplements, including enteral nutrition (EN) and total parental nutrition (TPN), have demonstrated to be effective in improving clinical outcomes and in decreasing the incidence of postoperative complications in major abdominal surgery [7,8], repairing immune function and reducing risk of sepsis, especially early EN [9,10].

Postoperative nutritional support can improve clinical outcome in patients underwent PD resolving the malnutrition status of the patients. Early EN after PD can influence both the endocrine and the exocrine function of the gland and can stimulate the pancreatic and bile secretion [11].

On the other hand, several studies reported different results and this is clear by reading the current nutritional guidelines. The current guidelines of the European Society for Parenteral and Enteral Nutrition recommend routine use of early enteral nutrition in patients undergoing major gastrointestinal surgery for cancer, including PD [12]. In contrast, the current American Society for Parenteral and Enteral Nutrition guidelines recommend postoperative nutritional support only in patients who are unlikely to meet their nutrient needs orally for a period of 7–10 days, which is not necessarily the case after PD [13]. Both of these guidelines are based on a few studies concerning pancreatic cancer.

The aim of this review is to focus on specific evidence regarding the enteral feeding strategy and the correlation between EN, main complications and outcomes after PD.

## 2. Enteral Nutrition and Outcomes after Pancreaticoduodenectomy

In 2006, Goonetilleke and Siriwardena published a systematic review regarding perioperative nutritional support after PD. The Authors examined 10 studies, investigating nutritional support after PD, and concluded favorably about EN, also showing that early mandatory, postoperative TPN was not associated with improved outcomes. The review of the Authors also implied that administration of postoperative EN helps to decrease infectious complications [14].

Further studies have been published after 2006, focusing on the relation between EN and postoperative complications, like delayed gastric emptying (DGE), postoperative pancreatic fistula (POPF), postpancreatectomy hemorrhage (PPH), infectious complications or clinical outcome, such as length of hospitalization.

### 2.1. Delayed Gastric Emptying (DGE)

Gastroparesis is one of the most important complications after PD, with a reported incidence between 19% and 44% even in centers dedicated to pancreatic surgery [15,16].

According to the International Study Group of Pancreas Surgery (ISGPS), delayed gastric emptying (DGE) is defined as the need of a nasogastric tube (NGT) for >3 days or the need of reinsert the NGT for persistent vomiting after surgery [17]. The ISGPS also classifies the severity of DGE as grade A: NGT required for 4–7 days or reinsertion after postoperative day (POD) 3 or inability to tolerate solid oral intake by POD 7; grade B: NGT required for 8–14 days or inability to tolerate solid oral intake by POD 14; grade C: NGT required for >14 days or inability to tolerate solid food by POD 21 [17].

The pathophysiological mechanism contributing to the development of gastroparesis is unknown. The mechanism is probably multifactorial. Likely factors involved in DGE after PD may be the presence of pancreatic fibrosis [18], intraperitoneal inflammation secondary to postoperative complications [19,20], gastrointestinal reconstruction [21,22], removal of the duodenum [23] or extended lymph node dissection [24,25]. Several authors suggest that DGE is also caused by gastric denervation due to the loss of parasympathetic nerves, resulting in the reduction of peristalsis and secretion of motilin [26]. Ligation of the right gastric and gastroduodenal arteries could be responsible for pylorus and antrum ischemia contributing to postoperative pylorospasm in pylorus-preserving PD [27]. When gastroparesis occurs it is very difficult to anticipate the need for nutritional support, but feeding can be administrated directly to the jejunum [28].

Some studies reported no increase or even decrease in the incidence of gastroparesis after PD with early EN [29]. Tien *et al.* performed a prospective randomized trial to test the effectiveness of biliopancraetic diversion with modified Roux-en-Y gastrojejunostomy reconstruction and of EN to minimize impacts of DGE after PD. In total 257 patients with periampullay tumors were randomized and underwent PD. DGE occurred in 20 patients (16.3%) in the EN group and 27 patients (21.7%) in the control group. Although statistical analysis revealed no difference (*p* = 0.27) in DGE between groups, ISGPS grades of gastric stasis were significantly lower in the modified group than in the control group (*p* = 0.001) [30].

In another randomized controlled trial, Mack *et al.* reported the benefit of gastric decompression and enteral feeding through a double-lumen gastrojejunostomy tube. Prolonged gastroparesis occurred in 4 controls (25%) and in none of the patients who had gastrojejunostomy (*p* = 0.03). In the author’s experience, the insertion of this kind of tube was found to be safe and feasible [31]. Although operative time was longer, no major problems occurred during tube insertion. Anyway, it is not clear if this result was due to the tube itself or to the efficacy of enteral nutrition.

In 2008 Grizas, comparing in a randomized controlled trial the effectiveness of the early enteral and natural nutrition after PD, did not notice any statistically significant difference in clinical or radiological manifestation of DGE between the two groups [32].

A recent meta-analysis of randomized controlled trial has been conducted to evaluate the safety and effectiveness of early enteral nutrition for patients after PD. Only four studies published in 2000 or later have been included in the analysis, involving 246 patients who underwent EN and 238 patients who underwent other nutritional routes. The rate of DGE in EN and other nutritional routes groups after PD was 15.9% and 18.9% respectively. Meta-analysis showed no statistically significant difference in DGE between EN and other nutritional routes group (OR, 0.89; 95% CI, 0.36–2.18; *p* = 0.79) [11]. Therefore, early EN would be tolerable for patients after PD, without increased gastroparesis. These results are in line with the previous studies regarding the effect of EN to DGE. However, this meta-analysis presents some limitations, due to the lack of relevant information in the original works. First of all, the definition of gastroparesis in the included studies was not always in according to DGE definition of ISGPS; the kind of tube placed in the jejunum and used for EN differed in the studies; the type of EN, cyclical or continuous, is important and it was not specified; van Berge Henegouwen *et al.* performed a randomized trial showing that in patients undergoing pylorus-preserving PD, the use of cyclical infusion of EN (stopped for 6 h overnight) had some advantages over continuous (24 h) EN [33]. Finally, the content of nutritional supplementation significantly influences the outcome. Gianotti *et al.*, performing randomized trial, reported that an enteral formula enriched with arginine, omega-3 fatty acids and RNA statistically reduced postoperative complications and length of hospitalization compared to standard enteral nutrition or TPN [34]. In addition, one of the studies in the meta-analysis did not report the nutritional route of the control group [31].

As reported, DGE is a multifactorial complication and still remains unclear which factors are related to gastroparesis. DGE incidence has been shown to be closely associated with postoperative pancreatic fistula, hemorrhage and abdominal collection [19]. In the study of Rayar *et al.*, protective factors reducing DGE were reported to be EN (*p* = 0.047, OR = 0.56) as well as the patient’s age (*p* < 0.01, OR = 1.01) and acute pancreatitis history prior to PD (*p* = 0.013, OR = 0.32) [35]. According to the authors, potential mechanisms of EN may be the mechanical effects of the nasojejunal tube across the anastomosis, which can stimulate the motility of the stomach and jejunum, or the stimulation of bowel peristalsis by the input of nutritional liquids [35,36].

It is clear therefore that even if some authors reported in previous study that EN was associated with a higher frequency of DGE [37], recent studies, especially randomized controlled trials, have shown that EN does not increase the rate of DGE [30,31,32,34]. Further studies and well-designed trials need to be conducted to demonstrate if EN is superior to early oral intake in preventing DGE after PD.

### 2.2. Postoperative Pancreatic Fistula

Postoperative pancreatic fistula (POPF) is another common complication in PD patients, with rates up to 12% in specialized centers [38].

According to ISGPS definition, POPF is defined as output via an operatively placed drain (or a subsequently placed percutaneous drain) of any measurable volume of drain fluid on or after postoperative day 3, with an amylase content greater than three times the upper serum value [39]. Okabayashi *et al.* investigating 100 patients who underwent PD from 1999 to 2007, identified, as independent parameter correlating with occurrence of POPF, not having early EN through the jejunostomy catheter (*p* = 0.007) [40]. In the series, this finding indicates that early EN was associated with a reduced incidence of fistula. The same Author, in a previous study, compared the outcome of patients underwent PD and were then either managed with early EN (starting on the day after surgery) or late EN (starting 7–14 days after surgery). In that study, early EN was associated not only with a decreased incidence of POPF, but also with sustained serum concentration of total protein and albumin, maintenance of body mass index, early restoration of peripheral lymphocyte count, and a shorter period of hospitalization [29].

In his retrospective study, Rayar did not detect any difference in the incidence of POPF between patients received EN until total oral alimentation compared to patients who did not receive EN and were orally fed after removing the nasogastric tube [35]. However, the incidence of POPF in control group may have been underestimated because routine amylase dosage in abdominal drain has only been performed since 2005.

Liu *et al.* observed a low rate of POPF in EN group compared to TPN group (*p* = 0.039). The reason of this difference remains unclear and requires further studies, hopefully with a major number of patients [41].

No difference in POPF rate has been reported in trials conducted comparing EN with other nutritional routes [30,31,32,42,43], even if only two studies reported the use of ISGPS definition of POPF [30,42]. Also, the meta-analysis of Shen, including POPF in intra-abdominal complication, showed no significant difference in intra-abdominal complications between EN and other nutritional routes [11]. However, in these studies EN appeared safe and tolerated for the patients after PD, even if it did not reveal any advantages in terms of POPF rate.

### 2.3. Postpancreatectomy Hemorrhage

Postpancreatectomy hemorrhage (PPH) is one of the most important complications after PD, with a reported incidence between 1% and 8% even in centers specializing in pancreatic surgery [44].

In 2007, the International Study Group of Pancreatic Surgery (ISGPS) proposed a new classification system of PPH with the aims to allow a proper diagnosis and to evaluate the severity of the hemorrhage; three different grades of PPH (grades A, B, and C) were defined according to the time of onset, site of bleeding, severity, and clinical impact [44]. As well known, PPH is also significantly related to many risk factors, such as POPF [45].

The retrospective study of Rayar reports a statistically significant difference in the incidence of PPH between the EN group and the control group (8% *vs.* 20%, respectively, *p* = 0.008). No significant differences were reported regarding grade and localization of the hemorrhage [35].

Concerning the protective factors for PPH, in 2011 Liu, comparing in randomized controlled trial the effectiveness of early enteral *vs.* total parental nutrition after PD, noticed that the incidence of PPH was significantly reduced in the EN group (1 case *vs.* 9 cases, respectively, *p* = 0.021) [41]. However the study was based on a small cohort (60 patients), and the inclusion criteria was so strict that most patients were in relatively good health conditions.

The meta-analysis of Shen reported only the rate of intra-abdominal complications, including also the PPH. Meta-analysis showed no statistically significant difference in intra-abdominal complications between EN and other nutritional routes (OR, 0.82; 95% CI, 0.53–1.26; *p* = 0.37) [11]. Due to the lack of relevant information in the original works, a subgroup analysis on PPH was not be performed; moreover, only one study used ISGPS’s definition to grade hemorrhage [30].

Regarding the influence of EN on the incidence of PPH, more studies are required, involving more patients and using the recommendations and the grades of ISGPS.

### 2.4. Length of Stay

One hundred and twenty-one patients with suspected operable upper gastrointestinal cancer (including 54 esophageal, 38 gastric and 29 pancreatic neoplasms) were randomized to receive early EN or control management (nil by mouth and IV fluids) in a prospective multicentre randomized controlled trial [43]. The results showed a clear advantage for EN in terms of length of stay (LOS) both intention-to-treat and per-protocol analysis (16 days *vs.* 19 days, *p* = 0.023 and *p* = 0.011, respectively) and there were no statistically significant differences between the two groups for hospital readmission after discharge. Even if it did not include only patients with pancreatic cancer, the importance of this study is that it represents the first adequately powered prospective randomized trial of early EN *vs.* “nil by mouth” in patients undergoing upper gastrointestinal resectional surgery in the United Kingdom.

Regarding the use of EN after PD to reduce the LOS, while several retrospective studies did not report any clear advantages compared to other nutritional route [35,46], Zhu *et al.* demonstrated a lower LOS combining EN with parental nutrition compared to TPN postoperative regimen (13.2 days *vs.* 16.8 days, *p* < 0.05) [47].

More evidence could be obtained from the meta-analysis of randomized controlled trial conducted by Shen [11]. Only two trials provided information regarding postoperative hospital stay, involving 388 patients [30,34]. EN was not associated with a significantly shorter LOS than other nutritional route (*p* = 0.74). As we have reported, this meta-analysis presents some limits, first of all, one of the two trials examined was published in 2000 [34], and several improvements have been obtained from 2000 to the present days regarding the postoperative management after PD. In addition, the trial of Tien included patients underwent biliopancreatic diversion with modified Roux-en-Y gastrojejunostomy reconstruction, so it is difficult to establish if the results are due to the modified surgical procedure or to the EN [30].

In a randomized controlled trial by Mack *et al.*, LOS was reduced by routine double-lumen gastrojejunostomy tube feeding compared to “standard care” (11.5 days *vs.* 15.8 days respectively, *p* = 0.01). However, it was not specified what the “standard care” meant, in fact patients in the control group were treated in according to the operating surgeon’s routine, including insertion of nasogastric tube and administration of nutritional support if the surgeon felt it was indicated, and the route of administration was also dictated by the surgeon’s routine practice [31]. It remains unclear how the results seen in the study were secondary to the tube itself or to the effects of EN. Furthermore, this study appeared to be underpowered (only 36 patients enrolled) to demonstrate improvements in outcomes typically studied in enteral feeding trial.

In the systematic review of Gerritsen *et al.*, including 15 studies of different feeding route after PD (7 randomized trials, 7 cohort studies and 1 case-control study), mean length of hospital stay was shorter in the oral diet and gastrojejunostomy tube groups, both at 15 days, followed by 19 days in jejunostomy tube, 20 days in the TPN and 25 days in the nasojejunal tube groups [48].

Several large studies found good results with a normal oral diet (without routine nutritional support) after PD. Yermilov *et al.* reviewed the California Cancer Registry (1194–2003) for outcomes of 1873 patients who underwent PD for adenocarcinoma receiving either parental feeding (14%), jejunostomy tube feeding (23%) or an oral diet without supplemental nutritional support (63%). They showed a significantly shorter LOS in the normal diet cohort [49].

A recent review suggested that implementation of a fast-track protocol, including early oral feeding, in pancreatic surgery could lead to reduce LOS and reduced costs without an increase in morbidity, mortality or readmission rates [50].

Future randomized studies should compare outcomes of a routine oral diet (with on demand nasojejunal feeding) with routine nasojejunal feeding after PD.

### 2.5. Infectious Complications

Several studies reported that the use of EN reduced the risk of infections. A meta-analysis by Braunschweig *et al.*, combining 27 randomized controlled trials (*n* = 1828), found a significantly lower risk of infections with enteral nutrition (RR = 0.64) compared to parental feeding. [51].

A systematic review and meta-analysis of randomized controlled trials comparing any type of enteral feeding started within 24 h after surgery with nil by mouth management in elective gastrointestinal surgery showed that early EN reduced the risk of any type of infection (relative risk 0.72, 95% confidence interval 0.54 to 0.98, *p* = 0.036) [52].

In the prospective randomized controlled trial by Barlow *et al.* of early EN *vs.* “nil by mouth” in patients undergoing upper gastrointestinal resectional surgery, operative morbidity was less common after early EN compared to control management (nil by mouth and IV fluids) (32.8% *vs.* 50.9 respectively, *p* = 0.044). In particular the study showed a significantly difference in the rate of wound and chest infections (*p* = 0.017 and *p* = 0.036, respectively) [43].

Even if these data suggest that the use of EN presents some advantages in terms of reduction of infectious complications, these studies did not include patients underwent only PD. Regarding the use of EN after PD, only two trials revealed advantages in terms of infectious complications [32,34]. Gianotti *et al.* performing randomized trial, reported that an enteral formula enriched with arginine, omega-3 fatty acids and RNA reduced statistically the infectious complications compared to TPN (8.4% *vs.* 22.1% respectively, *p* = 0.04) [34]. As well Grizas, comparing the effectiveness of the early enteral and natural nutrition regimen (from liquid to solid diet in the first five postoperative days) after PD, noticed a higher rate of postoperative complications in the natural nutrition group (53.3% *vs.* 23.3% respectively, *p* = 0.03) with an odds ratio of 3.8. This difference seems to occur due to higher incidence of infectious complications in the natural nutrition group (46.7% *vs.* 16.7% respectively, *p* = 0.025), with an odds ratio of 4.4 [32].

Hypothesis that EN is beneficial in reducing infectious complications is supported by the evidence that modifications in the mucosal defense have been implicated as important factors affecting infectious complications in critically ill patients [53]. EN influences the ability of gut-associated lymphoid tissue to maintain mucosal immunity. Both route and type of nutrition influence antibacterial respiratory tract immunity [54].

Despite these data, the recent meta-analysis of a randomized controlled trial by Shen *et al.* showed no significant difference in infections between early EN and other nutritional routes after PD [11].

Further and more powerful trials are probably required to understand the real benefit of EN in the reduction of infectious complications.

## 3. Conclusions

This review summarized the available evidence on early EN after PD, which appears to be safe and tolerated in patients but does not have clear advantages reducing DGE, POPF, PPH, infectious complications and LOS. Future large-scale, high-quality, multicenter trials are still required to clarify the role of early EN after PD.

## References

[1] Lewis S.J., Andersen H.K., Thomas S. (2009). Early enteral nutrition within 24 h of intestinal surgery *vs.* later commencement of feeding: A systematic review and meta-analysis. J. Gastrointest. Surg..

[2] Nygren J., Thorell A., Ljungqvist O. (2003). New developments facilitating nutritional intake after gastrointestinal surgery. Curr. Opin. Clin. Nutr. Metab. Care.

[3] Braga M., Ljungqvist O., Soeters P., Fearon K., Weimann A., Bozzetti F., ESPEN (2009). ESPEN Guidelines on Parenteral Nutrition: Surgery. Clin. Nutr..

[4] Wente M.N., Shrikhande S.V., Müller M.W., Diener M.K., Seiler C.M., Friess H., Büchler M.W. (2007). Pancreaticojejunostomy *vs.* pancreaticogastrostomy: Systematic review and meta-analysis. Am. J. Surg..

[5] Correia M.I., Caiaffa W.T., da Silva A.L., Waitzberg D.L. (2001). Risk factors for malnutrition in patients undergoing gastroenterological and hernia surgery: An analysis of 374 patients. Nutr. Hosp..

[6] Schnelldorfer T., Adams D.B. (2005). The effect of malnutrition on morbidity after Surgery for chronic pancreatitis. Am. Surg..

[7] Braga M., Vignali A., Gianotti L., Cestari A., Profili M., Carlo V.D. (1996). Immune and nutritional effects of early enteral nutrition after major abdominal operations. Eur. J. Surg..

[8] Ziegler T.R. (1996). Perioperative nutritional support in patients undergoing hepatectomy for hepatocellular carcinoma. JPEN J. Parenter. Enter. Nutr..

[9] Beier-Holgersen R., Boesby S. (1996). Influence of postoperative enteral nutrition on postsurgical infections. Gut.

[10] Slotwinski R., Olszewski W.L., Slotkowski M., Lech G., Zaleska M., Slotwinska S.M., Krasnodebski W.I. (2007). Can the interleukin-1 receptor antagonist (IL-1ra) be a marker of anti-inflammatory response to enteral immunonutrition in malnourished patients after pancreaticoduodenectomy?. J. Pancreas..

[11] Shen Y., Jin W. (2013). Early enteral nutrition after pancreatoduodenectomy: A meta-analysis of randomized controlled trials. Langenbecks Arch. Surg..

[12] Weimann A., Braga M., Harsanyi L., Laviano A., Ljungqvist O., Soeters P., Jauch K.W., Kemen M., Hiesmayr J.M., German Society for Nutritional Medicine (2006). ESPEN Guidelines on Enteral Nutrition: Surgery including organ transplantation. Clin. Nutr..

[13] ASPEN Board of Directors and the Clinical Guidelines Task Force (2002). Guidelines for the use of parenteral and enteral nutrition in adult and pediatric patients. J. Parenter. Enter. Nutr..

[14] Goonetilleke K.S., Siriwardena A.K. (2006). Systematic review of perioperative nutritional supplementation in patients undergoing pancreaticoduodenectomy. J. Pancreas..

[15] Qu H., Sun G.R., Zhou S.Q., He Q.S. (2013). Clinical risk factors of delayed gastric emptying in patients after pancreaticoduodenectomy: a systematic review and meta-analysis. Eur. J. Surg. Oncol..

[16] Yeo C.J., Cameron J.L., Sohn T.A., Lillemoe K.D., Pitt H.A., Talamini M.A., Hruban R.H., Ord S.E., Sauter P.K., Coleman J. (1997). Six hundred fifty consecutive pancreaticoduodenectomies in the 1990s: Pathology, complications, and outcomes. Ann. Surg..

[17] Wente M.N., Bassi C., Dervenis C., Fingerhut A., Gouma D.J., Izbicki J.R., Neoptolemos J.P., Padbury R.T., Sarr M.G., Traverso L.W. (2007). Delayed gastric emptying (DGE) after pancreatic surgery: A suggested definition by the International Study Group of Pancreatic Surgery (ISGPS). Surgery.

[18] Murakami H., Suzuki H., Nakamura T. (2002). Pancreatic fibrosis correlates with delayed gastric emptying after pylorus-preserving pancreaticoduodenectomy with pancreaticogastrostomy. Ann. Surg..

[19] Riediger H., Makowiec F., Schareck W.D., Hopt U.T., Adam U. (2003). Delayed gastric emptying after pylorus-preserving pancreatoduodenectomy is strongly related to other postoperative complications. J. Gastrointest. Surg..

[20] Park Y.C., Kim S.W., Jang J.Y., Ahn Y.J., Park Y.H. (2003). Factors influencing delayed gastric emptying after pylorus-preserving pancreatoduodenectomy. J. Am. Coll. Surg..

[21] Goei T.H., van Berge Henegouwen M.I., Slooff M.J., van Gulik T.M., Gouma D.J., Eddes E.H. (2001). Pylorus-preserving pancreatoduodenectomy: Influence of a Billroth I *vs.* a Billroth II type of reconstruction on gastric emptying. Dig. Surg..

[22] Takahata S., Ohtsuka T., Nabae T., Matsunaga H., Yokohata K., Yamaguchi K., Chijiiwa K., Tanaka M. (2002). Comparison of recovery of gastric phase III motility and gastric juice output after different types of gastrointestinal reconstruction following pylorus-preserving pancreatoduodenectomy. J. Gastroenterol..

[23] Müller M.W., Friess H., Beger H.G., Kleeff J., Lauterburg B., Glasbrenner B., Riepl R.L., Büchler M.W. (1997). Gastric emptying following pylorus-preserving whipple and duodenum-preserving pancreatic head resection in patients with chronic pancreatitis. Am. J. Surg..

[24] Yeo C.J., Cameron J.L., Sohn T.A., Coleman J., Sauter P.K., Hruban R.H., Pitt H.A., Lillemoe K.D. (1999). Pancreaticoduodenectomy with or without extended retroperitoneal lymphadenectomy for periampullary adenocarcinoma: Comparison of morbidity and mortality and short-term outcome. Ann. Surg..

[25] Yeo C.J., Cameron J.L., Lillemoe K.D., Sohn T.A., Campbell K.A., Sauter P.K., Coleman J., Abrams R.A., Hruban R.H. (2002). Pancreaticoduodenectomy with or without distal gastrectomy and extended retroperitoneal lymphadenectomy for periampullary adenocarcinoma, part 2: Randomized controlled trial evaluating survival, morbidity, and mortality. Ann. Surg..

[26] Tanaka A., Ueno T., Oka M., Suzuki T. (2000). Effect of denervation of the pylorus and transection of the duodenum on acetaminophen absorption in rats; possible mechanism for early delayed gastric emptying after pylorus preserving pancreatoduodenectomy. Tohoku J. Exp. Med..

[27] Kim D.K., Hindenburg A.A., Sharma S.K., Suk C.H., Gress F.G., Staszewski H., Grendell J.H., Reed W.P. (2005). Is pylorospasm a cause of delayed gastric emptying after pylorus-preserving pancreaticoduodenectomy?. Ann. Surg. Oncol..

[28] Kurahara H., Shinchi H., Maemura K., Mataki Y., Iino S., Sakoda M., Ueno S., Takao S., Natsugoe S. (2011). Delayed gastric emptying after pancreatoduodenectomy. J. Surg. Res..

[29] Okabayashi T., Kobayashi M., Nishimori I., Sugimoto T., Akimori T., Namikawa T., Okamoto K., Onishi S., Araki K. (2006). Benefits of early postoperative jejunal feeding in patients undergoing duodenohemipancreatectomy. World J. Gastroenterol..

[30] Tien Y.W., Yang C.Y., Wu Y.M., Hu R.H., Lee P.H. (2009). Enteral nutrition and biliopancreatic diversion effectively minimize impacts of gastroparesis after pancreaticoduodenectomy. J. Gastrointest. Surg..

[31] Mack L.A., Kaklamanos I.G., Livingstone A.S., Levi J.U., Robinson C., Sleeman D., Franceschi D., Bathe O.F. (2004). Gastric decompression and enteral feeding through a double-lumen gastrojejunostomy tube improves outcomes after pancreaticoduodenectomy. Ann. Surg..

[32] Grizas S., Gulbinas A., Barauskas G., Pundzius J. (2008). A comparison of the effectiveness of the early enteral and natural nutrition after pancreatoduodenectomy. Medicina.

[33] Van Berge Henegouwen M.I., Akkermans L.M., van Gulik T.M., Masclee A.A., Moojen T.M., Obertop H., Gouma D.J. (1997). Prospective randomized trial on the effect of cyclic *vs.* continuous enteral nutrition on postoperative gastric function after pylorus-preserving pancreatoduodenectomy. Ann. Surg..

[34] Gianotti L., Braga M., Gentilini O., Balzano G., Zerbi A., di Carlo V. (2000). Artificial nutrition after pancreaticoduodenectomy. Pancreas.

[35] Rayar M., Sulpice L., Meunier B., Boudjema K. (2012). Enteral nutrition reduces delayed gastric emptying after standard pancreaticoduodenectomy with child reconstruction. J. Gastrointest. Surg..

[36] Hallay J., Micskei C., Fülesdi B., Kovács G., Szentkereszty Z., Takács I., Sipka S., Bodolay E., Sápy P. (2008). Use of three lumen catheter facilitates bowel movement after pancreato-duodenectomy. Hepatogastroenterology.

[37] Martignoni M.E., Friess H., Sell F., Ricken L., Shrikhande S., Kulli C., Büchler M.W. (2000). Enteral nutrition prolongs delayed gastric emptying in patients after Whipple resection. Am. J. Surg..

[38] Alexakis N., Sutton R., Neoptolemos J.P. (2004). Surgical treatment of pancreatic fistula. Dig. Surg..

[39] Bassi C., Dervenis C., Butturini G., Fingerhut A., Yeo C., Izbicki J., Neoptolemos J., Sarr M., Traverso W., Buchler M. (2005). Postoperative pancreatic fistula: An international study group (ISGPF) definition. Surgery.

[40] Okabayashi T., Maeda H., Nishimori I., Sugimoto T., Ikeno T., Hanazaki K. (2009). Pancreatic fistula formation after pancreaticooduodenectomy; for prevention of this deep surgical site infection after pancreatic surgery. Hepatogastroenterology.

[41] Liu C., Du Z., Lou C., Wu C., Yuan Q., Wang J., Shu G., Wang Y. (2011). Enteral nutrition is superior to total parenteral nutrition for pancreatic cancer patients who underwent pancreaticoduodenectomy. Asia Pac. J. Clin. Nutr..

[42] Park J.S., Chung H.K., Hwang H.K., Kim J.K., Yoon D.S. (2012). Postoperative nutritional effects of early enteral feeding compared with total parental nutrition in pancreaticoduodectomy patients: A prosepective, randomized study. J. Korean Med. Sci..

[43] Barlow R., Price P., Reid T.D., Hunt S., Clark G.W., Havard T.J., Puntis M.C., Lewis W.G. (2011). Prospective multicentre randomised controlled trial of early enteral nutrition for patients undergoing major upper gastrointestinal surgical resection. Clin. Nutr..

[44] Wente M.N., Veit J.A., Bassi C., Dervenis C., Fingerhut A., Gouma D.J., Izbicki J.R., Neoptolemos J.P., Padbury R.T., Sarr M.G. (2007). Postpancreatectomy hemorrhage (PPH): An International Study Group of Pancreatic Surgery (ISGPS) definition. Surgery.

[45] Ricci C., Casadei R., Buscemi S., Minni F. (2012). Late postpancreatectomy hemorrhage after pancreaticoduodenectomy: Is it possible to recognize risk factors?. J. Pancreas.

[46] Gerritsen A., Besselink M.G., Cieslak K.P., Vriens M.R., Steenhagen E., van Hillegersberg R., Borel Rinkes I.H., Molenaar I.Q. (2012). Efficacy and complications of nasojejunal, jejunostomy and parenteral feeding after pancreaticoduodenectomy. J. Gastrointest. Surg..

[47] Zhu X.H., Wu Y.F., Qiu Y.D., Jiang C.P., Ding Y.T. (2013). Effect of early enteral combined with parenteral nutrition in patients undergoing pancreaticoduodenectomy. World J. Gastroenterol..

[48] Gerritsen A., Besselink M.G., Gouma D.J., Steenhagen E., Borel Rinkes I.H., Molenaar I.Q. (2013). Systematic review of five feeding routes after pancreatoduodenectomy. Br. J. Surg..

[49] Yermilov I., Jain S., Sekeris E., Bentrem D.J., Hines O.J., Reber H.A., Ko C.Y., Tomlinson J.S. (2009). Utilization of parenteral nutrition following pancreaticoduodenectomy: Is routine jejunostomy tube placement warranted?. Dig. Dis. Sci..

[50] Ypsilantis E., Praseedom R.K. (2009). Current status of fast-track recovery pathways in pancreatic surgery. J. Pancreas.

[51] Braunschweig C.L., Levy P., Sheean P.M., Wang X. (2001). Enteral compared with parenteral nutrition: A meta-analysis. Am. J. Clin. Nutr..

[52] Lewis S.J., Egger M., Sylvester P.A., Thomas S. (2001). Early enteral feeding *vs.* “nil by mouth” after gastrointestinal surgery: Systematic review and meta-analysis of controlled trials. BMJ.

[53] Li J., Kudsk K.A., Gocinski B., Dent D., Glezer J., Langkamp-Henken B. (1995). Effects of parenteral and enteral nutrition on gut-associated lymphoid tissue. J. Trauma..

[54] King B.K., Kudsk K.A., Li J., Wu Y., Renegar K.B. (1999). Route and type of nutrition influence mucosal immunity to bacterial pneumonia. Ann. Surg..

